# Trauma-induced Acute Epidural Hematoma: The Rising Sun in a Progressively Lethargic Man

**DOI:** 10.7759/cureus.3162

**Published:** 2018-08-20

**Authors:** Christ Ordookhanian, Paul E Kaloostian

**Affiliations:** 1 School of Medicine, University of California, Riverside, USA; 2 Neurological Surgery, University of California Riverside School of Medicine, Riverside, USA

**Keywords:** accident, edh, epidural, extradural, hematoma, hemorrhage, traumatic brain injury

## Abstract

A young adult, 18 years of age, presented to the emergency department with severe traumatic brain injury (TBI) resulting from a bicycle versus vehicle head-on collision. The patient initially presented in a promising condition but quickly deteriorated into a state of unconsciousness with no meaningful responses to stimuli or coordinated voluntary movement. Stat computed tomography (CT) revealed a large, right-sided, acute epidural hematoma (EDH) with mass-effect and a severe midline shift indicative of immediate surgery. This case highlights the importance of closely monitoring traumatic brain injury patients regardless of initial presentation and neurological exam results, as the patient's condition may drastically and rapidly change without much warning. Additionally, it is key to utilize regular radiological studies on these patients, to detect any neurological changes as close to onset as possible. Lastly, it is imperative that neurosurgeons closely monitor the patients/ state of consciousness as a rapid decline serves as a key diagnostic indicator of the need for immediate surgery.

## Introduction

Acute complications of traumatic brain injury (TBI) should be a major concern for neurosurgeons worldwide, as the delayed onset of an epidural hematoma, although rare, can occur, resulting in the rapid decline of a patient’s condition [1]. A large-population case study conducted by Irie et al. in 2011 revealed that individuals aged 10 to 24 typically acquired epidural hematomas as a result of motor vehicle accidents. Additionally, 81% of the total epidural hematomas result from accidental injury. In addition, 75% of the study population also had a varying severity of skull fracture [2]. When evaluating TBI patients, the possibility of delayed onset epidural hematoma (EDH) should be floating in the midst of a neurosurgeon’s mind. Factors such as age, the severity of the TBI, and neurological status are key diagnostic parameters that impact the risk of developing a delayed-onset epidural hematoma. Routine CT radiological imaging every four to six hours is essential for detecting delayed EDH onset, while quite easy to do so, overlooking the parameters we discussed could position the patient down an undesirable route that may lead to morbidity and/or mortality [3].

## Case presentation

**Figure 1:**
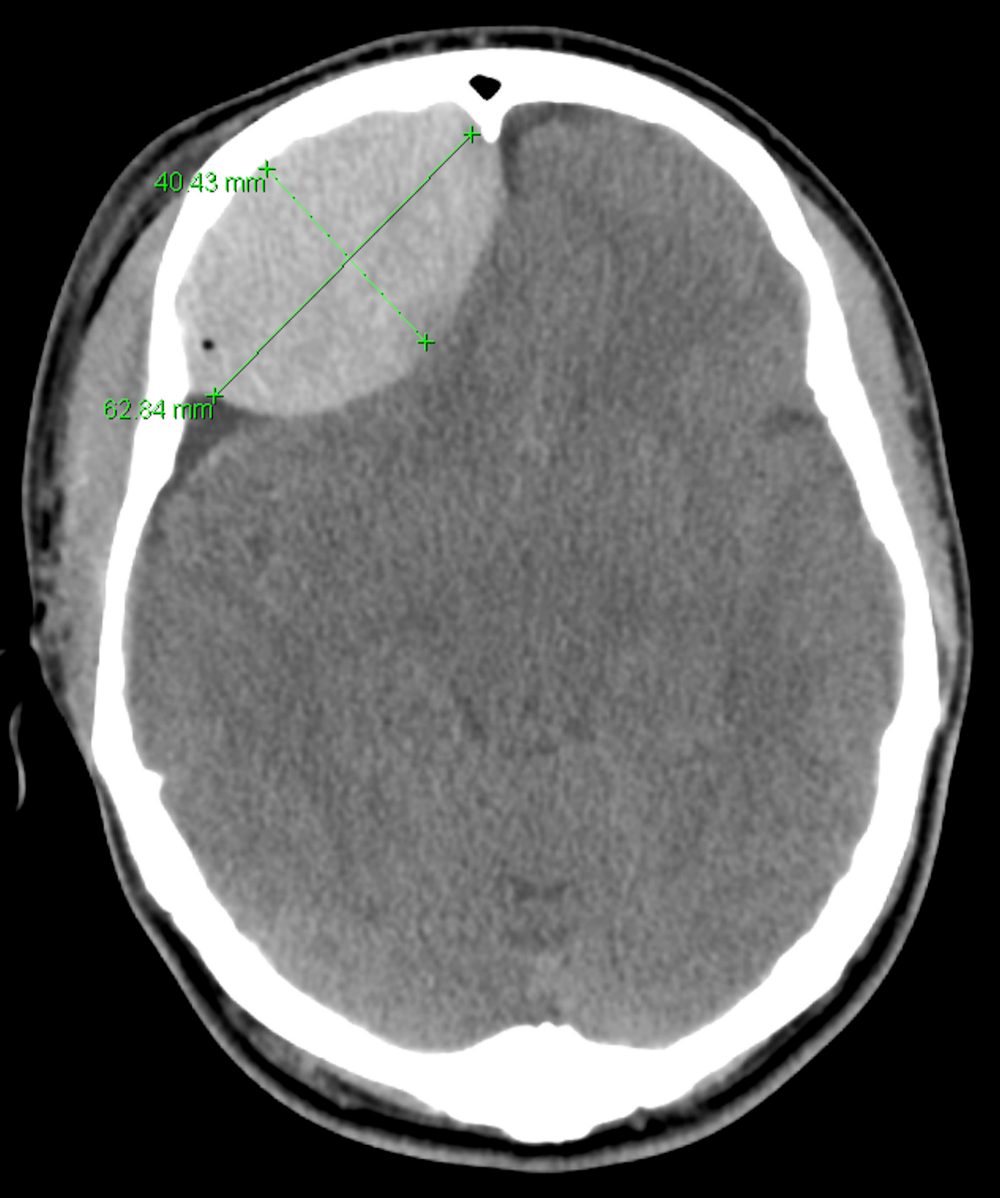
Computed tomography (CT) scan of an 18-year-old bicycle versus vehicle head-on patient Computed tomography (CT) study conducted on an 18-year-old male patient upon the immediate deterioration of the initial presenting condition (GCS 13 to GCS 5 in four hours). This image highlights the presence of a large, acute, right-sided EDH, measuring 62.84 mm by 40.43 mm, as well as a 6-mm midline shift. The patient underwent an immediate craniotomy where the entirety of the EDH was removed. Intraoperatively, a nondisplaced frontotemporal skull fracture was noted. The patient responded positively to the surgical treatment and was discharged on postoperative day four with a complete resolution of the initial and developed conditions. EDH: epidural hematoma

A Hispanic male, 18 years of age, presented to the emergency department (ED) after a head-on collision with a vehicle traveling at 30 miles per hours (mph) while riding his bicycle. Our patient initially presented in a promising condition with a Glasgow Coma Score (GCS) of 13, however, this rapidly deteriorated to a GCS of 5 within a four-hour window. Immediate CT studies (Figure 1) revealed the presence of a large, right-sided, acute EDH with a 6-mm midline shift, indicating the need for immediate surgical intervention.

An emergent right craniotomy for acute EDH evacuation was performed, where, intraoperatively, a nondisplaced frontotemporal skull fracture was noted with bleeding from the branches of the middle meningeal artery, which was immediately stopped. Postoperatively, the patient’s condition improved from a pre-operative GCS of 5 to a postoperative GCS of 15. Our patient was hospitalized for 72 hours and released on postoperative day four with a follow-up scheduled for two weeks later. The patient was recommended to complete a four-week course of physical and occupational therapy to regain full pre-traumatic injury quality of life. 

## Discussion

The ideal management of traumatic brain injury (TBI) patients has been a neglected subset of neurological surgery and emergency medicine for much of allosteric medicinal practices since its existence. Recent advancements in medicinal technology, specifically high-resolution radiological imaging, has opened the door for novel pathology discovery techniques. Patients presenting with any trauma to the head should be subjected to a thorough neurological examination by a neurosurgeon or neurologist to ensure the intactness of the nervous system. However, the management of TBI patients is not a static task; constant monitoring of the patient’s condition and re-evaluation is of key importance. In our case, an 18-year-old male initially presented with a GCS of 13 but within a mere four hours, there was a rapid deterioration to a GCS of 5, a state of coma and unconsciousness. The rapid deterioration of a patient’s condition following a TBI serves as a highly reliable diagnostic tool for the need for immediate radiological studies and, often, surgical intervention. Upon our patient's rapid deterioration, a stat CT scan was ordered where a large, right-sided, acute EDH, with a 6-mm midline shift was identified. Patients with delayed onset epidural hematomas are also more likely to have an overlying skull fracture. Through stat emergency department imaging studies, no skull fracture was visualized, however, intraoperatively, a nondisplaced frontotemporal skull fracture was noted with bleeding. This highlights a key concept that radiological imaging is not a concrete diagnostic tool and serves as an aid to our professional opinion. Upon the completion of craniotomy, the patient made a full, expected recovery and was discharged on postoperative day four. Patients with traumatic brain injury are unique and, often, high-demand patients who require constant monitoring and advanced care. Ignoring the symptoms of any complexity may become deadly in a short period of time and greatly reduce the chances of a complete postoperative outcome. Throughout the years, EDH recovery rates have improved greatly and, today, only bear a two to five percent mortality rate, thanks to the improved care of TBI patients from the emergency department, neurosurgery, and allied healthcare team members.

## Conclusions

The imperative findings of this report highlight how essential the rapid detection of delayed onset EDH is in saving a life. Letting the sun fully rise may lead to not so bright times. Serial radiological imaging is crucial for TBI patients in identifying developing neurologically relevant pathology; paying close attention to the condition of the patient is a key diagnostic tool. Any enlarging EDH with mass-effect on the brain constitutes the need for immediate surgical intervention to maximize post-traumatic outcomes.
